# Comparative Secretomics Analysis Reveals the Major Components of *Penicillium oxalicum* 16 and *Trichoderma reesei* RUT-C30

**DOI:** 10.3390/microorganisms9102042

**Published:** 2021-09-27

**Authors:** Kexin Wang, Nian Zhang, Robin Pearce, Shi Yi, Xihua Zhao

**Affiliations:** 1 College of Life Science, Jiangxi Normal University, Nanchang 330022, China; wkx15170407885@163.com (K.W.); zn1656706745@163.com (N.Z.); m18270850038@163.com (S.Y.); 2 Department of Computational Medicine and Bioinformatics, University of Michigan, Ann Arbor, MI 48109, USA; robpearc@umich.edu; 3 Nanchang Key Laboratory of Microbial Resources Exploitation & Utilization from Poyang Lake Wetland, Jiangxi Normal University, Nanchang 330022, China

**Keywords:** secretome, *Penicillium oxalicum* 16, *Trichoderma reesei* RUT-C30, major components

## Abstract

In this study, the major secretome components of *Penicillium oxalicum* 16 and *Trichoderma reesei* RUT-C30 under wheat bran (WB) and rice straw (RS) solid-state fermentation were systematically analyzed. The activities of the major components, e.g., cellulase, hemicellulase, and amylase, were consistent with their abundance in the secretomes. *P. oxalicum* 16 secreted more abundant glycoside hydrolases than *T. reesei* RUT-C30. The main up-regulated proteins from the induction of WB, compared with that from RS, were amylase, pectinase, and protease, whereas the main down-regulated enzymes were cellulase, hemicellulase, swollenin, and lytic polysaccharide monooxygenase (LPMO). Specifically, WB induced more β-1,4-glucosidases, namely, S8B0F3 (UniProt ID), and A0A024RWA5 than RS, but RS induced more β-1,4-exoglucanases and β-1,4-endoglucanases, namely, A0A024RXP8, A024SH76, S7B6D6, S7ZP52, A024SH20, A024S2H5, S8BGM3, S7ZX22, and S8AIJ2. The *P. oxalicum* 16 xylanases S8AH74 and S7ZA57 were the major components responsible for degrading soluble xylan, and S8BDN2 probably acted on solid-state hemicellulose instead of soluble xylan. The main hemicellulase component of *T. reesei* RUT-C30 in RS was the xyloglucanase A0A024S9Z6 with an abundance of 16%, but *T. reesei* RUT-C30 lacked the hemicellulase mannanase and had a small amount of the hemicellulase xylanase. *P. oxalicum* 16 produced more amylase than *T. reesei* RUT-C30, and the results suggest amylase S7Z6T2 may degrade soluble starch. The percentage of the glucoamylase S8B6D7 did not significantly change, and reached an average abundance of 5.5%. The major auxiliary degradation enzymes of *P. oxalicum* 16 were LPMOs S7Z716 and S7ZPW1, whereas those of *T. reesei* RUT-C30 were swollenin and LPMOs A0A024SM10, A0A024SFJ2, and A0A024RZP7.

## 1. Introduction

Carbohydrates are the most widely distributed, abundant renewable biomass resource on Earth [1,2]. Improper treatment such as in situ incineration of large amounts of idle carbohydrates causes environmental pollution and resource waste [2]. In addition, non-renewable resources, such as fossil energy and lithium for batteries, which are extremely popular at present, will eventually be exhausted [3,4,5]. Therefore, it is of great value to transform superfluous carbohydrates into useful biofuels and chemicals [6].

The most abundant carbohydrates include cellulose, which constitutes 40–60% of the total biomass on Earth [7], hemicellulose which accounts for 20–35% of the renewable resources [8], and the widely distributed starch. The corresponding enzymes that hydrolyze these three abundant carbohydrates are cellulase, hemicellulase, and amylase, respectively. Cellulase is further classified into three classes, i.e., β-1,4-endoglucanases (EG; EC 3.2.1.4), cellobiohydrolases (CBH; EC 3.2.1.91), and β-1,4-glucosidases (BGL; EC 3.2.1.21) [9]. Hemicellulase includes xylanase (EC 3.2.1.8), mannanase (EC 3.2.1.78) and xyloglucanase (EC 3.2.1.151) [10]. Amylase is composed of α-amylase (EC 3.2.1.1), β-amylase (EC 3.2.1.2), and glucoamylase (EC 3.2.1.3) [11]. In addition to these enzymes, lytic polysaccharide monooxygenase (LPMO) participates in oxidative degradation of solid carbohydrates (cellulose, starch, hemicellulose, chitin, etc.) [12,13,14] and swollenin accelerates the expansion and fracture of solid cellulose [15].

In previous studies, we screened a new, fast-growing wild fungus, *Penicillium oxalicum* 16, which secretes amylase, cellulase, and hemicellulase [2,16], and the cellulase combination from *P. oxalicum* 16 and *Trichoderma reesei* RUT-C30 can synergistically degrade cellulose [2]. Although some studies have sequenced and analyzed the genomes and secretomes of *P. oxalicum* and *T. reesei* [10,17,18,19,20,21], and concluded that *P. oxalicum* has more diverse lignocellulolytic enzymes according to genomics information, particularly for cellulose binding domain-containing proteins and hemicellulases compared to the widely used cellulase producer *T. reesei* [20], the main components have not been systematically and completely summarized. Furthermore, we found that, compared to *T. reesei* RUT-C30, the yield of cellulase in *P. oxalicum* 16 is not sufficiently high [2,16]. Therefore, it is important to quantitate the secretome information of *P. oxalicum* 16 before engineering this useful strain.

WB and RS are the main agricultural wastes in China. In our previous study, we found that wheat bran (WB) and rice straw (RS) compositions are very different: WB is made of 36% cellulose, 28% hemicellulose, 8% lignin, 5% ash, 12% starch, and 11% other components, including pectin and protein, and RS comprises about 30% cellulose, 25% hemicellulose, 19% lignin, 18% ash, and 8% other constituents [2].

Although the enzymes and BGL of *P. oxalicum* 16 were studied by our group [2,4,5,22], its secretomics information remains unknown. To address this, here we investigated the main components by comparative secretomics analysis of *P. oxalicum* 16 and *T. reesei* RUT-C30 through WB or RS solid-state fermentation. The results of the analysis provide different conclusions from previous reports, such as showing lower hemicellulase of *P. oxalicum* 16 compared to other studies [10,20,21], in addition to revealing amylase information, among other novel findings.

## 2. Materials and Methods

### 2.1. Materials

*P. oxalicum* 16 was deposited in the China Center for Type Culture Collection (CCTCC, Wuhan, China) with the accession number AF2015017, and *T. reesei* RUT-C30 was obtained from the New World Institute of Biotechnology.

Carboxymethylcellulose sodium salt (CMC), 4-nitrophenyl-β-D-cellobioside (pNPC), xylan, NH_4_HCO_3_, dithiothreitol, iodoacetamide, and salicin were purchased from Sigma-Aldrich (St. Louis, MO, USA). Soluble starch and microcrystalline cellulose (MCC) were purchased from Sinopharm Chemical Reagent Co., Ltd. (Beijing, China). Trypsin, formic acid, and acetonitrile were purchased from Promega (Madison, WI, USA), Sigma-Aldrich Fluka (St. Louis, MO, USA), and Fisher Chemical (Fair Lawn, NJ, USA), respectively.

### 2.2. Enzyme Production and Extraction

Approximately, 2 × 10^6^ spores of *T. reesei* RUT-C30 and *P. oxalicum* 16 were incubated in 250 mL Erlenmeyer flasks with the solid-state medium containing either 5 g of WB or RS, 0.09 g KH_2_PO_4_, 0.09 g (NH_4_)_2_SO_4_, 0.015 g CaCl_2_, 0.015 g urea, 0.015 g MgSO_4_·7H_2_O, and 200 μL Mandels mineral salt solution [2,23]. The solid-state mediums inoculated with the two strains were cultured at 75% humidity, an initial pH of 5, and were kept at 28 °C for 5 days.

To extract enzymes, the suspension containing 1 g of dry solid-state medium and 3 mL of acetate buffer (50 mM, pH 5) was shaken at 180 rpm at room temperature for 1 h, the supernatant was collected by centrifugation for 10 min at 10,000× *g* at 4 °C, and the precipitate was resuspended with 3 mL of acetate buffer twice.

### 2.3. Determination of Enzyme Activity and Protein Content

The enzyme activities of EG, BGL, xylanase, and amylase were assayed using the dinitrosalicylic acid (DNS) method [2,24]. Specifically, 50 μL diluted culture supernatants were mixed with 450 μL of 1% of the corresponding substrates CMC, salicin, xylan, and soluble starch (50 mM acetate buffer, pH 5) at 50 °C for 30 min, and the reaction was stopped by adding 500 μL DNS. Then the mixture was boiled for 10 min and cooled on ice to stabilize the color. One enzyme activity unit was defined as the amount of enzyme that produced 1 μmol reducing sugar (i.e., glucose or xylose) within a minute at the given experimental conditions.

CBH’s activity was determined by releasing 4-nitrophenol measured at 420 nm after adding 150 μL of 10% Na_2_CO_3_ to stop the reaction. A quantity of 100 μL diluted culture supernatants was incubated with 50 μL of 1 mg/mL pNPC (50 mM acetate buffer, pH 5) at 50 °C for 30 min [16,25]. One CBH activity unit was defined as the amount of enzyme which released 1 μmol 4-nitrophenol per minute.

Protein was quantified by the Bradford method using bovine serum albumin as a standard [26].

### 2.4. Pretreatment of Corncob Powder (Pr-CP), RS (Pr-RS), and MCC (Pr-MCC)

RS was cut to about 3 cm, and milled by machine (Huangcheng 800, Yongkang, China) for 5 min, which was not filtered using any mesh sieve. CP and milled RS were pretreated using a laboratory autoclave (Boxun 18 L, Shanghai, China) at 121 °C for 1 h, and then washed with deionized water until the washed water was sugar-free and the pH was 7. All Pr-CP and Pr-RS were dried at 55 °C to a constant weight and subsequently milled to 100 mesh particle size for further use.

To prepare Pr-MCC, 3 g cellufloc-200 cellulose, 20 glass balls with 3 mm diameters, and 60 mL deionized water were added to a 250 mL Erlenmeyer flask, put into a laboratory autoclave (Boxun 18 L, Shanghai, China) at 121 °C for 30 min, and then shaken at 180 rpm for 48 h. Pr-MCC was washed 3 times and dried at 55 °C to a constant weight for further use.

### 2.5. Enzymatic Hydrolysis of Pr-CP, Pr-RS, and Pr-MCC

A quantity of 25 mg of Pr-CP, Pr-RS, or Pr-MCC was incubated with 100 μg of the cultured supernatant enzymes from *T. reesei* RUT-C30 or *P. oxalicum* 16 with a total water-insoluble solid loading of 2.5% (*w/v*) (the final volume was 1 mL). The hydrolytic reactions were carried out in 50 mM acetate buffer (pH 5) at 50 °C and 180 rpm for 96 h; the total sugar produced in the reactions was determined by the anthrone colorimetric method at 600 nm [27]. All reactions were carried out in a 1.5 mL EP tube.

### 2.6. SDS-PAGE of Secretome, and In-Gel Digestion

To obtain 50 μg of protein in the separating gel of SDS-PAGE, electrophoresis was carried out at 120 V for 40 min, and the gel stained by Coomassie blue was decolorized. Gel pieces that could not be decolorized were cut from the decolorized gel for further use.

We sent gel pieces to PTM BioLab Inc. (Hongzhou City, China), for identification of the secretomes. For in-gel tryptic digestion, gel pieces were destained in the solution containing 50 mM NH_4_HCO_3_ and 50% acetonitrile (*v/v*) until clear [28]. Gel pieces were dehydrated with 100 μL of 100% acetonitrile for 5 min. Then the liquid was removed, and the gel pieces were rehydrated in 10 mM dithiothreitol and incubated at 56 °C for 60 min. Following this, the gel pieces were again dehydrated in 100% acetonitrile; after the liquid was removed, the gel pieces were rehydrated with 55 mM iodoacetamide. Samples were incubated at room temperature in the dark for 45 min. Gel pieces were washed with 50 mM NH_4_HCO_3_ and dehydrated with 100% acetonitrile. Gel pieces were rehydrated with 10 ng/μL trypsin resuspended in 50 mM NH_4_HCO_3_ on ice for 1 h. Excess liquid was removed, and gel pieces were digested with trypsin at 37 °C overnight. Peptides were extracted with 50% acetonitrile/5% formic acid and followed by 100% acetonitrile. Peptides were dried to completion and resuspended in 2% acetonitrile/0.1% formic acid.

### 2.7. LC-MS/MS Analysis

The tryptic peptides were dissolved in solvent A (0.1% formic acid) and directly loaded onto a reversed-phase analytical column (15 cm × 75 μm) made by PTM BioLab Inc. The gradient for solvent B (0.1% formic acid in 98% acetonitrile) was from 6 to 23% for 16 min, 23 to 35% for 8 min, and climbed to 80% for 3 min, and was finally kept at 80% for 3 min. A constant flow rate of 400 nL/min was used in the EASY-nLC 1000 UPLC system.

The peptides were subjected to NSI source followed by tandem mass spectrometry (MS/MS) in Q ExactiveTM Plus (Thermo, Waltham, MA, USA) coupled online to the UPLC [29]. The electrospray voltage applied was 2.0 kV. The m/z scan range was 350 to 1800 for a full scan, and intact peptides were detected in the Orbitrap at a resolution of 70,000. Peptides were then selected for MS/MS using an NCE set at 28 and the fragments were detected in the Orbitrap at a resolution of 17,500. A data-dependent procedure that alternated between one MS scan followed by 20 MS/MS scans with 15.0 s dynamic exclusion was used. Automatic gain control (AGC) was set at 5E4.

### 2.8. Data Processing

The resulting MS/MS data were processed using Proteome Discoverer 1.3. Tandem mass spectra were searched against the UniProt *P. oxalicum* database (9977 sequences) and UniProt *T. reesei* database (9848 sequences) (https://www.uniprot.org, accessed on 24 March 2017), and the Mycocosm database (https://mycocosm.jgi.doe.gov/ accessed on 4 September 2021). The annotation of substrate of lignocellulase was manually predicted by combination of UniProt, Mycocosm, Baidu Search, and NCBI Blast. Trypsin/P (or other enzymes if any) was specified as a cleavage enzyme allowing up to two missing cleavages. The mass error was set to 10 ppm for precursor ions and 0.02 Da for fragment ions. The carbamidomethyl of Cys was specified as a fixed modification, whereas oxidation of Met was specified as a variable modification. Peptide confidence was set as “high” and the peptide ion score was set to >20.

## 3. Results

### 3.1. Enzymatic Activities of Major Glycoside Hydrolases (GHs)

Various GHs of *P. oxalicum* 16 cultured in the WB solid-state medium (16WB), *P. oxalicum* 16 cultured in the RS solid-state medium (16RS), *T. reesei* RUT-C30 cultured in the WB solid-state medium (C30WB), and *T. reesei* RUT-C30 cultured in the RS solid-state medium (C30RS) were induced, and their enzymatic activities were determined. As shown in Table 1, 16WB achieved the highest activities for a number of enzymes, e.g., 998 IU/gds amylase, 283 IU/gds xylanase, and 42 IU/gds BGL, but it produced the lowest activities for EG and CBH. 16RS attained increased EG and CBH activities of 211 and 0.31 IU/gds, respectively, but its amylase and xylanase activities were only 373 and 150 IU/gds, respectively. C30RS showed the highest activities of EG and CBH, which were approximately three and 21 times higher than those of 16WB. Although C30WB EG activity was slightly higher than that of 16WB and 16RS, its amylase and xylanase activities were about 100 and 3.6 times lower than those of 16WB.

### 3.2. Hydrolytic Ability of GHs

To evaluate the degradation ability of GHs from 16WB, 16RS, C30WB, and C30RS toward pretreated agricultural waste, we measured the total sugar released from WB, Pr-MCC, Pr-RS, and Pr-CP. The chemical compositions of WB and RS without pretreatment were introduced in our previous study [2]; MCC is pure cellulose, and the composition of CP contains 35.1–35.87% cellulose, 34.1–34.4% xylan, 20.9–21.96% lignin, and 8.1–9.6% other components [30,31]. Thus, Pr-RS and Pr-CP are intermediate biomasses between WB and Pr-MCC. Therefore, these chemical compositions contribute to our understanding of biomass degradation. As shown in Table 2, 16WB produced the highest percentage of total sugar with 10,571 μg/mL when hydrolyzing WB, which was 1.67, 5.43, and 7 times higher than 16RS, C30WB, and C30RS, respectively. The total sugar released from Pr-RS had the lowest percentage compared with that from WB, Pr-MCC, and Pr-RS, indicating that Pr-RS was the most difficult to degrade. Additionally, when considering Pr-MCC and Pr-CP, C30RS produced more total sugar than C30WB, 16RS, and 16WB.

### 3.3. Percent Abundance of the Identified Proteins

To obtain the GH distribution, it was important to analyze the secretomes of the four enzymatic preparations. The number of identified proteins for 16RS and 16WB reached 291, 181 proteins of which were quantified. In contrast, 532 proteins were identified for C30RS and C30WB, among which 100 proteins were quantified. The total number of GH families (GHFs) from the 16WB secretome reached 88 and was 1.42, 1.76, and 3.14 times higher than that of 16RS, C30WB, and C30RS, respectively, indicating that 16WB produced more abundant and extensive GHs as seen in the Supplementary Materials.

**Figure 1:**
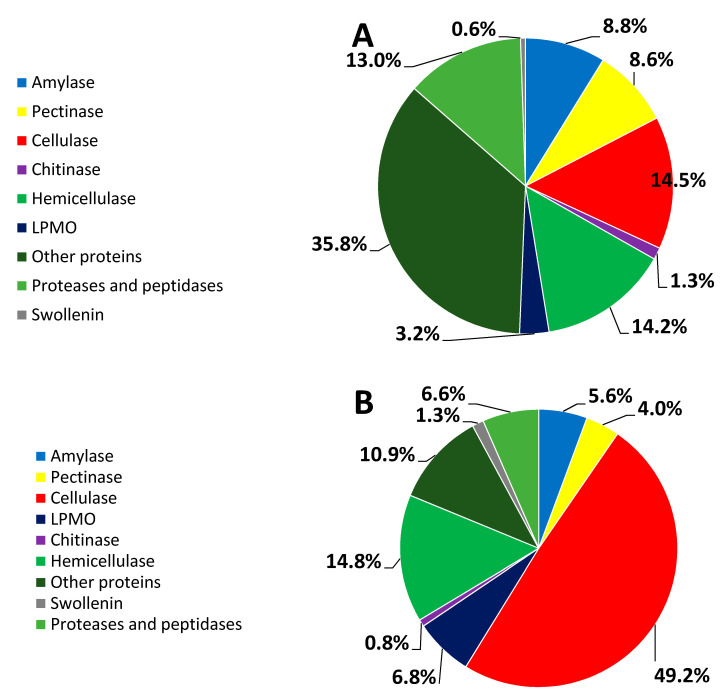
Percentage of the proteins identified in *P. oxalicum* 16 ((**A**): 16WB; (**B**): 16RS).

As shown in Figure 1A, 35.8% of the secretome of 16WB was composed of other proteins such as 0.09% laccase which degrades lignin. The 16WB hemicellulase accounted for 14.2% of all the identified proteins. The other enzymes that had a relatively high percentage (e.g., >8%) were protease and peptidase (13%), cellulase (14.5%), amylase (8.8%), and pectinase (8.6%), which includes rhamnogalacturonan proteins, arabinofuranosidase, arabinosidase, arabinanase, endo-polygalacturonase, and pectin lyase. In contrast to the low expression of cellulase in 16WB, the 16RS had a high percentage of cellulase, up to 49.2% (Figure 1B). The percentage of the 16RS hemicellulase was similar to that of the 16WB hemicellulase (14.2% vs. 14.8%). The auxiliary enzymes for degrading cellulose mainly included LPMO and swollenin, which showed a higher percentage in 16RS than in 16WB.

**Figure 2:**
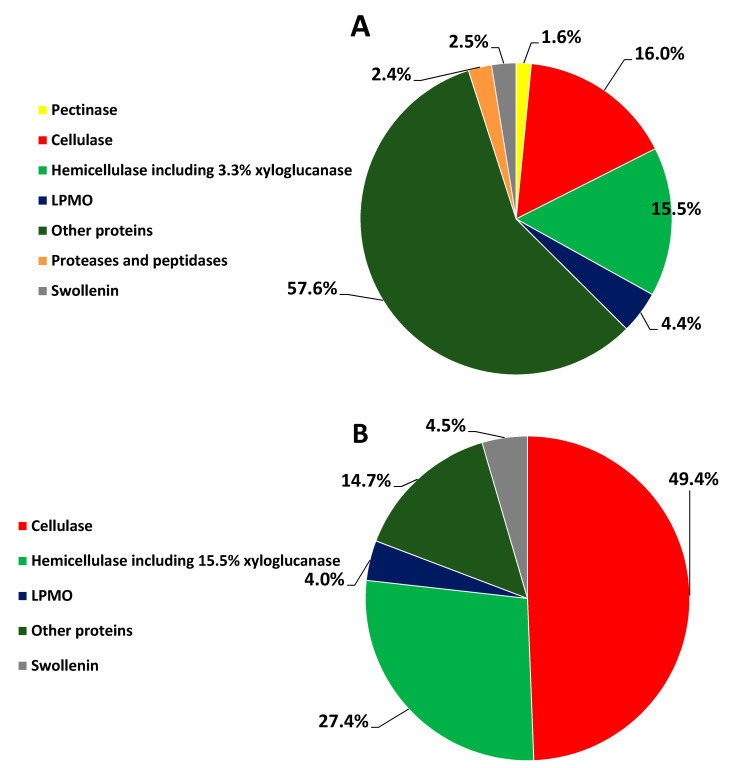
Percentage of the proteins identified in *T. reesei* Rut-C30 ((**A**): C30WB; (**B**): C30RS).

As shown in Figure 2A, C30WB produced 1.6% pectinase (arabinofuranosidase), 57.6% other proteins with 10.5% oxidoreductase, 16% cellulase, and 15.5% hemicellulase including 3.3% xyloglucanase. In addition, C30WB secreted 2.5% swollenin, and 2.4% proteases and peptidases. As shown in Figure 2B, C30RS produced 49.4% cellulase, 27.4% hemicellulase including 15.5% xyloglucanase, and 4.5% swollenin, which was 3.5 times higher than in 16RS.

As shown in Figure 1 and Figure 2, C30RS and 16RS strongly produced cellulase, hemicellulase, swollenin, and LPMO, surpassing C30WB and 16WB. Chitinase and amylase were produced by 16WB and 16RS, but they did not exist in either C30WB or C30RS. The protease and peptidase percentages of 16WB and 16RS was higher than those of C30WB, and C30RS did not possess proteases or peptidase. Moreover, 16RS and 16WB hemicellulases had lower percentages than those of C30WB and C30RS, but the 16RS and 16WB GHs were more balanced than C30WB and C30RS as described above, consistent with a previous study [10].

**Figure 3:**
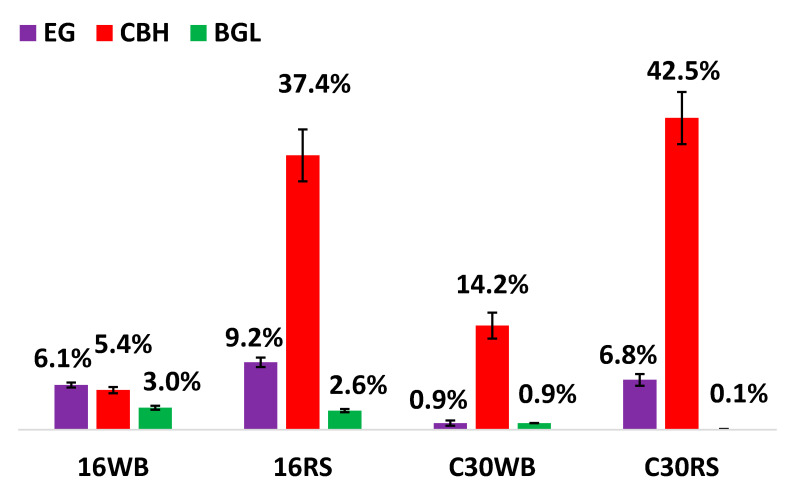
Percentage of EG, CBH, and BGL.

Figure 3 shows the percentage of CBH, EG, and BGL from 16WB, 16RS, C30RS, and C30WB. 16WB CBH and EG had the lowest percentage, but it obtained the highest percentage of BGL (~3%). The C30RS and 16RS achieved the highest percentages of CBH at 42.5% and 37.4%, respectively. In addition, it was obvious that C30WB and C30RS had a very low percentage of BGL, suggesting that *T. reesei* RUT-C30 may be a poor expressor of BGL.

### 3.4. Revealing Up-Regulated and Down-Regulated Proteins

As shown in Table 3 and Table 4, the up-regulated proteins of 16WB compared with 16RS were mainly related to amylase, pectinase, and protease, whereas the down-regulated proteins of that were mainly related to cellulase, hemicellulase, swollenin, and LPMO. Compared with the 16RS secretome, the up-regulated classification of the main 16WB components consisted of 30 proteins, i.e., putative EG (UniProt ID S7ZMB4), α-amylase (S7Z6T2), glucoamylase (S7ZIW0), glycosidase (S8AIA9), α-1,2-mannosidase (S7Z4H1), α-galactosidase (S7ZFY8), β-mannosidase A (S8BFI1), endo-polygalacturonase (S7ZD03), β-xylanase (S7ZA570, S7ZAV8), endo-β-1,4-mannanase F (S7ZDN1), endo-1,3-β-glucanase eglC (S7ZAG7), non-reducing end α-L-arabinofuranosidase (S7ZW00), CBH I (S7ZJL3), arabinogalactan endo-β-1,4-galactanase (S7ZBM4), β-galactosidase (S7Z5H6), putative endo-β-1,4-xylanase (S8AH74), chitinase (S8AWH6), putative α-L-arabinofuranosidase (S7Z4P2, S8B8M7), putative rhamnogalacturonan α-L-rhamnopyranohydrolase (S7ZZQ8), putative β-glucuronidase (S8B0N0), putative exo-β-1,3-galactanase (S7ZUD9), putative β-glucanase (S7ZMU5), putative β-1,3-1,4-glucanase (S7ZCP1), putative endo-β-1,4-xylanase (S8B2H7, S8AH74), putative α-mannosidase (S8B2R2, S8AUX2), and putative endo-β-1,6-galactanase(S8AXM3); the down-regulated classification of the main 16WB components including auxiliary decomposed enzymes of cellulose was comprised of 19 proteins, namely, putative β-1,6-glucanase (S8AMF6), putative endo-β-1,3-glucanase (S7ZAS9), CBH I (S7ZRD6), CBH II (S7ZP52), endo-1,4-β-xylanase (S8AMN0, S8BDN2), glucoamylase (S8B6D7), chitinase (S7Z8G1, S7ZR03, and S8B6N1), BGL (S8B0F3), non-reducing end α-L-arabinofuranosidase (S7Z3I8), endo-β-1,4-mannanase F (S7ZL65), EG (S8BGM3, S7ZX22, and S8AIJ2), EG1 (S8B2B2), putative swollenin (S7ZAB6), and LPMO (S7ZPW1).

Compared with the C30RS secretome, the up-regulated classification of the main C30WB components included 13 proteins, which were β-mannosidase (A0A024RUF8), endo-polygalacturonase (A0A024S1V1), β-1,4-endoxylanase (A0A024S0A7), endo-β-1,6-galactanase (A0A024S0G1), xylanase (A0A024RWW9), β-1,3-endoglucanase (A0A024S1W9), α-galactosidase (A0A024SGF7), α-glucuronidase (A0A024S166), β-xylosidase (A0A024SDM6), GH16 domain-containing protein (A0A024S732), chitinase (A0A024S1T5), α-L-arabinofuranosidase (A0A024S2Y7), and β-1,3-endoglucanase (A0A024SAF4); the down-regulated classification of the main components including auxiliary decomposed enzymes of cellulose from the C30WB secretome was comprised of 15 proteins which were xyloglucanase (A0A024S9Z6), EG (A0A024SNB7, A0A024SH20, A0A024S2H5), CBH I (A0A024RXP8), CBH II (A0A024SH76), non-reducing end α-L-arabinofuranosidase (A0A024SGE7), endo-1,4-β-xylanase (P36217, A0A024SIB3), chitinase (A0A024S0K1), mannan endo-1,4-β-mannosidase (A0A024SIJ3), β-1,4-endoxylanase (A0A024RV01), BGL (A0A024SCX9), LPMO (A0A024SFJ2), and swollenin (A0A024RZP7).

### 3.5. Ascertaining Identities with a High Percentage

Revealing the identities of the highly expressed GHs is helpful to design GH systems that are suitable for diverse substrates. As shown in Table 5, β-xylanase (S7ZA57) ranked first in 16WB with an abundance of 6.5%, glucoamylase (S8B6D7) ranked second at 5.7%, and CBH I (S7ZRD6) ranked third at 3.7%. EG S8AH74 and hemicellulase endo-β-1,4-mannanase F (S7ZDN1) obtained abundances of 1.1% and 1.7%, respectively, but they decreased sharply in RS. 16RS CBH I (S7B6D6) had the highest abundance at 26%, whereas the second-highest was β-xylanase (S8BDN2) at 14% in 16RS, but the 16RS β-xylanases S7ZA57 and S8AH74 decreased to 0.36% and 0.06%, respectively. 16RS CBH II (S7ZP52) possessed the third-highest percentage at 9.6%. Of particular interest was that glucoamylase (S8B6D7) also achieved an amazing 5.2% in 16RS. In addition, three EGs (S8BGM3, S7ZX22, and S8AIJ2) of 16RS also increased significantly to 3.7%, 3.3%, and 1.2%, respectively. The BGL (S8B0F3) percentage of 16WB and 16RS changed little and accounted for 3 and 2.5%, respectively. According to our secretomics information, we studied *P. oxalicum* 16 BGL (S8B0F3) [4,5,22], and its properties were improved by directed evolution [4,5]. The main LPMO was S7ZPW1, and reached 5.6% in 16RS and 3.2% in 16WB, but the main LPMO S7Z716 only existed in 16RS at an abundance of 1.2%.

CBH I removes cellobiose from the reducing end of the cellooligosaccharide, whereas CBH II releases cellobiose from the non-reducing end of the cellooligosaccharide. In general, these two enzymes work together to accelerate the degradation of cellulose. As shown in Table 5, the average percentage of CBH I and CBH II from 16RS and 16WB was 2.7:1, but that of CBH I and CBH II from C30RS and C30WB was close to 1:1. Both of the C30RS CBH I (A0A024RXP8) and CBH II (A0A024SH76) percentages were 21% and were 2.68 and 3.39 times higher, respectively, than those from C30WB. C30RS xyloglucanase (A0A024S9Z6) accounted for 16%, which was 4.7 times higher than C30WB. However, xyloglucanase was not found in 16WB and 16RS. There was only one glucoamylase (A0A024SN40) in *T. reesei* RUT-C30, but we could not find it in C30RS or C30WB. Other hydrolases may have taken the place of the amylase role for hydrolyzing starch, but the degradation efficiency was extremely low. In addition, the percentage of the swollenin (A0A024RZP7) from C30RS was 4.5% and was 1.8 times higher than that from C30WB.

## 4. Discussion

Natural substrates generate more GHs than pure substrates for fungi [10,32,33,34], so we used WB and RS for induction, rather than MCC, CMC, xylan, or starch. In addition, WB and RS are the main agricultural wastes in the north and south of China, respectively, so it was meaningful to realize utilization of “turning waste into treasure”. WB mainly consists of cellulose, hemicellulose, starch, and a small amount of pectin, whereas RS is mainly composed of cellulose, hemicellulose, and lignin [2]. In our study, WB induced more diverse secretome proteins than RS and is suitable for inducing pectinase, xylanase, and amylase; however, RS generated more cellulase, hemicellulase, LPMO, and swollenin. Some studies have shown that lactose, sophorose, or sophorose analogs were the main inducers of *T. reesei* RUT-C30 [34], but they are expensive and not suitable for industrial application. However, WB and RS are a good choice as cheap and widely existing carbon sources.

Designing enzyme preparation according to different substrates is beneficial and effective to completely degrade the renewable biomass resources with their complex and stubborn structures [10,19,20]. Therefore, it is important to thoroughly ascertain the secretome information of *P. oxalicum* 16 and *T. reesei* RUT-C30, which will lay a foundation for future molecular modification. Through our comparative analysis, we found that the *P. oxalicum* 16 xylanases S8AH74 and S7ZA57 are probably the main factors for the degradation of soluble xylan. However, the increased percentage of the xylanase S8BDN2 did not improve the hydrolysis of soluble xylan, but on the contrary, its degradation ability of soluble xylan was reduced. Therefore, we believe that it is highly likely that S8BDN2 acts on solid-state hemicellulose instead of soluble xylan. Furthermore, we speculate that the amylase S7Z6T2 is the main degradation factor for soluble starch, because the glucoamylase S8B6D7 did not significantly increase or decrease under the induction of WB or RS. We could not find amylase or glucoamylase in C30WB or C30RS, indicating that *T. reesei* RUT-C30 is not an amylase producer.

Natural cellulose is the most abundant renewable biomass resource, but it is difficult to degrade [35]. Therefore, how to effectively degrade cellulose is an ongoing key issue, and it has been proven that its degree of decomposition is mainly related to CBHs [36]. In addition, according to comparative secretomics analysis of the two strains, we found that the highest yield of secreted enzymes from the induction of RS was that of CBH. Thus, we focused on the discussion concerning the synergistic degradation of cellulose. The whole enzymatic system of 16RS and C30RS showed a significant difference in the hydrolysis of Pr-MCC and Pr-CP. Although CBH I was strongly induced in 16RS, the total proportion of CBH I S7ZRD6 and CBH II S7ZP52 (about 2.7:1) in 16RS exceeded that of C30RS. Furthermore, the proportion of CBH I A0A024RXP8 and CBH II A0A024SH76 in C30RS was close to 1:1. Therefore, the different proportions of CBH I and CBH II in 16RS and C30RS may have resulted in the different hydrolysis degrees of Pr-MCC and Pr-CP (Table 2), which is consistent with the findings reported by Schülein et al. [36]. Different combinations and proportions of CBH I, CBH II, and EG may or may not produce synergistic degradation against different substrates [36]. It has been demonstrated that the substrates CMC or MCC do not trigger synergistic degradation by CBH I and EG, but amorphous cellulose leads to synergistic degradation [36]. CBH I and EG with a proportion of 1:1 have the greatest synergistic degradation, but CBH II and EG show synergistic degradation against any substrate [34]. CBH I and CBH II, with a ratio of 1:4, exhibits the maximum synergistic degradation of solid cellulose [34].

In contrast to GHs, the auxiliary degradation enzyme LPMO, which is a kind of metal enzyme containing Cu^2+^ and generates a synergistic degradation effect with cellulase, amylase, hemicellulase, etc., uses cellobiose deoxygenase and ascorbic acid as electron donors, and the peroxide hydrogen and oxygen as co-substrates [12,13]. In the study, LPMO A0A024SM10 was only secreted in C30WB, but not in C30RS. LPMOs S7ZPW1 and A0A024SFJ2 were up-regulated in 16RS and C30RS, respectively. Our inference that the main substrate of A0A024SM10 and A0A024SFJ2 is cellulose is consistent with the report of Corrêa et al. [14], and other substrates of A0A024SM10 may be pectin or hemicellulose. LPMO S7Z716 only existed in 16RS, and could not be detected in 16WB. The relative abundance of S7ZPW1 was increased in 16RS. In addition, we found that RS had more cellulose, whereas WB had more starch, pectin, etc. in our previous work [2]. Therefore, we speculated that S7Z716 and S7ZPW1 may mainly perform oxidative cleavage of cellulose. Although reducing sugar could not be detected in the reaction solution, swollenin can indeed break and expand crystalline cellulose [15]. Interestingly, the LPMO in 16RS showed a higher relative abundance than that in C30RS, but swollenin in *T. reesei* RUT-C30 had a higher relative abundance than that in *P. oxalicum* 16 (Table 5). Additionally, C30RS enzymes released more reduced sugar when dealing with Pr-MCC (Table 2). Therefore, we speculate that the C30RS swollenin goes beyond its LPMO and assumes the main responsibility of synergistic degradation against cellulose.

An effective cellulase preparation with the proportion 4:1:1 of CBH II, CBH I, and EG, with the addition of swollenin and LPMO, will be the focus of our future work. According to the above results, *P. oxalicum* 16 possesses more diverse GHs than *T. reesei* RUT-C30, so engineering the strain *P. oxalicum* 16 will be used to generate the proportion with auxiliary degradation enzymes according to its genomics, transcriptomics and secretomics information. By analogy, other effective enzyme mixtures can be also created.

## 5. Conclusions

The secretome comparison analysis here revealed that WB induced *P. oxalicum* 16 and *T. reesei* RUT-C30 to produce more abundant and balanced GHs than RS, but RS induced more cellulase and hemicellulase. Moreover, the study found that *P. oxalicum* 16 possessed more diverse GHs than *T. reesei* RUT-C30. In addition, the study characterized the up-regulated, down-regulated, and main component identities. Based on the study, it may be feasible to design combinations from the modified *P. oxalicum* 16 to decompose specific substrates.

## Figures and Tables

**Table 1:** Comparation of the main GH activities.

Origins	EG (IU/gds)	CBH (IU/gds)	BGL (IU/gds)	Amylase (IU/gds)	Xylanase (IU/gds)
16WB	209 ± 2	0.02 ± 0.0	42 ± 1.3	998 ± 8	283 ± 2
16RS	211 ± 2	0.31 ± 0.01	32.1 ± 1.2	373 ± 1	150 ± 3
C30WB	259 ± 1	0.09 ± 0.00	9.2 ± 1.0	24.8 ± 1.1	73 ± 0
C30RS	628 ± 4	0.41 ± 0.02	11.0 ± 0.8	10.2 ± 0.3	79 ± 6

The experiments were repeated three times.

**Table 2:** Total soluble sugars released from pretreated agricultural wastes for 96 h.

Origins	Pr-RS (μg/mL)	WB (μg/mL)	Pr-MCC (μg/mL)	Pr-CP (μg/mL)
16WB	1821 ± 72	10,571 ± 478	3475 ± 63	4101 ± 63
16RS	3488 ± 81	6322 ± 441	4625 ± 370	8278 ± 171
C30WB	3209 ± 480	1948 ± 424	6107 ± 117	7978 ± 111
C30RS	3650 ± 153	1509 ± 43	7222 ± 73	8674 ± 189

The experiments were repeated three times.

**Table 3:** The up-regulated and down-regulated proteins of 16WB compared with 16RS.

UniProt ID	Protein Description	Substrate	Classification	16WB/16RS Ratio	Regulated Type
S8AHA8	Putative β-xylosidase	xylooligosaccharide	GH3	168 ± 8	Up
S8BFI1	Putative β-mannosidase	β-mannose-oligosaccharide	GH2	71 ± 7	Up
S7ZIW0	Glucoamylase	dextrin	GH15	38 ± 1	Up
S7Z5H6	β-galactosidase	β-lactose	GH35	29 ± 1	Up
S7ZA57	β-xylanase	xylan	GH10	10 ± 0	Up
S8B2R2	Putative α-mannosidase	α-mannose-oligosaccharide	GH92	10 ± 1	Up
S8AH74	Putative endo-β-1,4-xylanase	xylan	GH30	10 ± 1	Up
S7ZW00	Putative α-L-arabinofuranosidase	arabinogalactan, arabinoglycan, etc.	GH62	9.4 ± 0.4	Up
S8AXM3	Putative endo-β-1,6-galactanase	β-1,6-galactan	GH30	9.2 ± 0.8	Up
S7ZUD9	Putative exo-β-1,3-galactanase	β-1,3-galactan	GH43	7.3 ± 0.6	Up
S7ZBM4	Arabinogalactan endo-β-1,4-galactanase	β-galactosidic linkages in type I arabinogalactans	GH53	7.1 ± 0.4	Up
S8B8M7	Putative α-L-arabinofuranosidase	arabinogalactan, arabinoglycan, etc.	GH43	6.6 ± 0.2	Up
S7ZMB4	Putative EG	cellulose	GH12	5.1 ± 0.1	Up
S8B2H7	Putative endo-β-1,4-xylanase	xylan	GH30	4.5 ± 0.3	Up
S7ZJL3	CBH I	MCC	GH7	3.0 ± 0.1	Up
S8B0N0	Putative β-glucuronidase	proteoglycan	GH2	2.8 ± 0.2	Up
S7ZMU5	Putative β-glucanase	β-1,3(4)-glucan	GH16	2.4 ± 0.1	Up
S7ZFY8	α-galactosidase	α-lactose	GH27	2.2 ± 0.1	Up
S8AWH6	Putative chitinase	chitin	GH18	1.7 ± 0.1	Up
S7Z6T2	α-amylase Amy13A	α-1,4-starch	GH13	1.7 ± 0.1	Up
S7ZZQ8	Putative α-L-rhamnopyranohydrolase	R-α-L-rhamnopyranoside	GH28	1.7 ± 0.1	Up
S7ZD03	Endo-polygalacturonase	pectin or pectinic acid	GH28	1.6 ± 0.1	Up
S7Z4P2	Putative α-L-arabinofuranosidase	arabinogalactan, arabinoglycan, etc.	GH43	1.6 ± 0.1	Up
S7Z4H1	α-1,2-mannosidase	α-1,2-mannose-oligosaccharide	GH47	1.6 ± 0.1	Up
S7ZAV8	β-xylanase	xylan	GH10	1.5 ± 0.0	Up
S8B7P9	Putative α-L-arabinofuranosidase	arabinogalactan, arabinoglycan, etc.	GH54	1.5 ± 0.1	Up
S7ZWC7	Putative exo-α-L-1,5-arabinanase	α-L-1,5-arabinoglycan	GH93	1.5 ± 0.1	Up
S7ZDN1	Putative endo-β-1,4-glucanase	cellulose	GH5	1.4 ± 0.1	Up
S7ZCP1	Putative β-1,3-1,4-glucanase	β-1,3-1,4-glucan	GH16	1.4 ± 0.1	Up
S8AUX2	Putative α-mannosidase	α-mannose-oligosaccharide	GH92	1.3 ± 0.1	Up
S7ZR03	Putative chitinase	chitin	GH18	0.76 ± 0.04	Down
S8B6D7	Glucoamylase	dextrin	GH15	0.70 ± 0.03	Down
S8B0F3	BGL	cellooligosaccharide	GH3	0.67 ± 0.03	Down
S7Z8G1	Putative chitinase	chitin	GH18	0.54 ± 0.03	Down
S8AMF6	Putative β-1,6-glucanase	β-1,6-glucan	GH30	0.53 ± 0.01	Down
S8B6N1	Putative chitinase	chitin	GH18	0.51 ± 0.01	Down
S8AXN0	Putative pectate lyase	pectinic acid	polysaccharide lyase 1 family	0.43 ± 0.00	Down
S7Z3I8	Putative α-L-arabinofuranosidase	arabinogalactan, arabinoglycan, etc.	GH62	0.40 ± 0.02	Down
S7ZPW1	LPMO	polysaccharide	AA9	0.33 ± 0.00	Down
S7ZAS9	Putative endo-β-1,3-glucanase	β-1,3-glucan	Pectate lyase superfamily	0.32 ± 0.00	Down
S7ZAB6	Putative swollenin	solid cellulose	Expansin_EG45	0.24 ± 0.01	Down
S7ZP52	CBH II	MCC	GH6	0.09 ± 0.00	Down
S8B2B2	EG1	cellulose	GH7	0.09 ± 0.00	Down
S7ZL65	Putative β-1,4-mannanase	mannan	GH5	0.08 ± 0.00	Down
S8AMN0	Endo-1,4-β-xylanase	xylan	GH11	0.08 ± 0.00	Down
S7ZRD6	CBH I	MCC	GH7	0.08 ± 0.00	Down
S8BGM3	EG	cellulose	GH5	0.06 ± 0.00	Down
S8BDN2	β-xylanase	xylan	GH10	0.04 ± 0.00	Down
S7ZX22	EG	cellulose	GH5	0.04 ± 0.00	Down
S8AIJ2	EG	cellulose	GH5	0.02 ± 0.00	Down

The experiments were repeated three times. 16WB/16RS ratio > 1 is defined as up-regulation, and 16WB/16RS ratio < 1 is defined as down-regulation.

**Table 4:** The up-regulated and down-regulated proteins of C30WB compared with C30RS.

UniProt ID	Protein Description	Substrate	Classification	C30WB/C30RS Ratio	Regulated Type
A0A024SGF7	α-galactosidase	α-lactose	GH27	12 ± 0	Up
A0A024S1T5	Chitinase	chitin	GH18	7.7 ± 0.7	Up
A0A024SDM6	β-xylosidase	xylooligosaccharide	GH3	5.9 ± 0.4	Up
A0A024RWW9	xylanase	xylan	GH30	5.8 ± 0.3	Up
A0A024SAF4	β-1,3-endoglucanase	β-1,3-glucan	GH17	4.0 ± 0.1	Up
A0A024S166	α-glucuronidase	xylan	GH67	3.3 ± 0.1	Up
A0A024S732	β-glucanase	β-1,3(4)-glucan	GH16	2.5 ± 0.2	Up
A0A024S1W9	β-1,3-endoglucanase	β-1,3-glucan	GH17	2.3 ± 0.1	Up
A0A024S0A7	β-1,4-endoxylanase	xylan	GH43	1.9 ± 0.1	Up
A0A024S2Y7	α-N-arabinofuranosidase	α-L-arabinoside	GH54	1.9 ± 0.1	Up
A0A024S1V1	Endopolygalacturonase	pectin, pectinic acid	GH28	1.8 ± 0.1	Up
A0A024RUF8	β-mannosidase A	β-mannose-oligosaccharide	GH2	1.5 ± 0.1	Up
A0A024S0G1	Endo-β-1,6-galactanase	β-1,6-galactan	GH30	1.2 ± 0.1	Up
A0A024SIJ3	β-mannase (Fragment)	mannan	GH5	0.62 ± 0.02	Down
A0A024RZP7	Swollenin	solid cellulose	Expansin_EG45	0.50 ± 0.03	Down
A0A024SNB7	EG	cellulose	GH7	0.49 ± 0.02	Down
P36217	Endo-1,4-β-xylanase 2	xylan	GH11	0.46 ± 0.01	Down
A0A024RXP8	CBH I	MCC	GH7	0.33 ± 0.01	Down
A0A024S0K1	Chitnase	chitin	GH18	0.33 ± 0.02	Down
A0A024SGE7	α-L-arabinofuranosidase	α-L-arabinoside	GH62	0.30 ± 0.00	Down
A0A024SH76	CBH II	MCC	GH6	0.26 ± 0.01	Down
A0A024S9Z6	Xyloglucanase	xyloglucan	GH74	0.22 ± 0.00	Down
A0A024SFJ2	LPMO	polysaccharide	AA9	0.21 ± 0.00	Down
A0A024RV01	β-1,4-endoxylanase	xylan	GH30	0.16 ± 0.01	Down
A0A024SH20	EG	cellulose	GH5	0.15 ± 0.00	Down
A0A024SCX9	BGL	cellooligosaccharide	GH3	0.10 ± 0.00	Down
A0A024SIB3	Endo-1,4-β-xylanase 3	xylan	GH10	0.09 ± 0.00	Down
A0A024S2H5	EG	cellulose	GH12	0.05 ± 0.00	Down

The experiments were repeated three times. C30WB/C30RS ratio > 1 is defined as up-regulation, and C30WB/C30RS ratio <1 is defined as down-regulation.

**Table 5:** Identities of the main components.

Origins	UniProt ID	Description	Relative Abundance (%)	Substrate	Classification
16WB	S7ZRD6	CBH I	3.7 ± 0.2	MCC	GH7
	S7ZP52	CBH II	1.5 ± 0.0	MCC	GH6
	S7ZA57	β-xylanase	6.5 ± 0.3	xylan	GH10
	S8AH74	endo-β-1,4-xylanase	1.1 ± 0.0	xylan	GH30
	S8B6D7	Glucoamylase	5.7 ± 0.3	dextrin	GH15
	S7Z6T2	α-amylase my13A	1.8 ± 0.1	starch	GH13
	S7ZDN1	Endo-β-1,4-mannanase F	1.7 ± 0.0	mannan	GH5
	S7ZMB4	EG	1.5 ± 0.0	cellulose	GH12
	S8B0F3	BGL	3.0 ± 0.1	cellooligosaccharide	GH3
	S7ZPW1	LPMO	3.2 ± 0.1	polysaccharide	AA9
	S7ZAB6	Swollenin	0.6 ± 0.0	solid cellulose	Expansin_EG45
16RS	S7ZRD6	CBH I	26 ± 3	MCC	GH7
	S7ZP52	CBH II	9.6 ± 0.6	MCC	GH6
	S8BDN2	β-xylanase	14 ± 1	xylan	GH10
	S8B6D7	Glucoamylase	5.2 ± 0.3	dextrin	GH15
	S7Z6T2	α-amylase my13A	0.7 ± 0.0	starch	GH13
	S8BGM3	EG	3.7 ± 0.1	cellulose	GH5
	S7ZX22	EG	3.3 ± 0.1	cellulose	GH5
	S8AIJ2	EG	1.2 ± 0.0	cellulose	GH5
	S8B0F3	BGL	2.5 ± 0.1	cellooligosaccharide	GH3
	S7Z716	LPMO	1.2 ± 0.0	polysaccharide	AA9
	S7ZPW1	LPMO	5.6 ± 0.2	polysaccharide	AA9
	S7ZAB6	Swollenin	1.3 ± 0.1	solid cellulose	Expansin_EG45
C30WB	A0A024RXP8	CBH I	7.8 ± 0.4	MCC	GH7
	A0A024SH76	CBH II	6.2 ± 0.3	MCC	GH6
	A0A024S9Z6	Xyloglucanase	3.3 ± 0.0	xyloglucan	GH74
	A0A024RWA5	BGL	0.5 ± 0.2	cellooligosaccharide	GH3
	A0A024SM10	LPMO	3.4 ± 0.2	polysaccharide	AA9
	A0A024SFJ2	LPMO	1.0 ± 0.1	polysaccharide	AA9
	A0A024RZP7	Swollenin	2.5 ± 0.1	solid cellulose	Expansin_EG45
C30RS	A0A024RXP8	CBH I	21 ± 2	MCC	GH7
	A0A024SH76	CBH II	21± 1	MCC	GH6
	A0A024S9Z6	Xyloglucanase	16 ± 1	xyloglucan	GH74
	A0A024SH20	EG	5.5 ± 0.4	cellulose	GH5
	A0A024S2H5	EG	1.2 ± 0.0	cellulose	GH12
	A0A024SFJ2	LPMO	4.0 ± 0.1	polysaccharide	AA9
	A0A024RZP7	Swollenin	4.5 ± 0.2	solid cellulose	Expansin_EG45

The experiments were repeated three times.

## Supplementary Materials

The following are available online at www.mdpi.com/article/10.3390/microorganisms9102042/s1, Excel Table S1: Identified protein information.
